# The Impact of a Conditional Cash Transfer on Multidimensional Deprivation of Young Women: Evidence from South Africa’s HTPN 068

**DOI:** 10.1007/s11205-020-02367-y

**Published:** 2020-06-09

**Authors:** Kelly Kilburn, Lucia Ferrone, Audrey Pettifor, Ryan Wagner, F. Xavier Gómez-Olivé, Kathy Kahn

**Affiliations:** 1 Carolina Population Center, University of North Carolina, Chapel Hill, NC USA; 2 Duke Global Health Institute, Duke University, Durham, NC USA; 3 Department of Economics and Management, University of Florence, via delle Pandette, 9, 50127 Florence, Italy; 4 Department of Epidemiology, University of North Carolina, Chapel Hill, NC USA; 5 Wits Reproductive Health and HIV Institute, University of the Witwatersrand, Johannesburg, South Africa; 6 MRC/Wits Rural Public Health and Health Transitions Research Unit (Agincourt), School of Public Health, Faculty of Health Sciences, University of the Witwatersrand, Johannesburg, South Africa; 7 Umeå Centre for Global Health Research, Division of Epidemiology and Global Health, Department of Public Health and Clinical Medicine, Umeå University, Umeå, Sweden; 8 INDEPTH Network, Accra, Ghana

**Keywords:** South Africa, Multidimensional poverty, Cash transfer, Young women, I32, I31, 015

## Abstract

Despite the growing popularity of multidimensional poverty measurement and analysis, its use to measure the impact of social protection programs remains scarce. Using primary data collected for the evaluation of HIV Prevention Trials Network (HPTN) 068, a randomized, conditional cash transfer intervention for young girls in South Africa that ran from 2011 to 2015, we construct an individual-level measure of multidimensional poverty, a major departure from standard indices that use the household as the unit of analysis. We construct our measure by aggregating multiple deprivation indicators across six dimensions and using a system of nested weights where each domain is weighted equally. Our findings show that the cash transfer consistently reduces deprivations among girls, in particular through the domains of economic agency, violence, and relationships. These results show how social protection interventions can improve the lives of young women beyond single domains and demonstrate the potential for social protection to simultaneously address multiple targets of the SDGs.

## Introduction

The multidimensional nature of wellbeing and poverty has long been recognized in the social sciences. The psychological concept of subjective wellbeing is multidimensional, composed of several domains (Headey et al. 1985; Diener 1984), while the concept of multidimensional deprivation in economics is closely related to Sen’s Capability Approach and its subsequent developments (Sen 1979, 1981). This approach views poverty as more than lack of monetary means but rather a lack of realization and fulfillment of one’s potential (Sen 1985, 1999).

One of the earlier attempts to quantify human progress in more comprehensive ways than economic growth was made by Terleckyj (1970), who proposed a framework and methodology to measure social progress at local and national levels, using twenty indicators such as life-expectancy and people with disabilities. In the 1970s, the literature that conceptualized poverty in terms broader than monetary means began to grow and focused heavily on measuring basic needs. Hicks and Streeten (1979) argued that national GDP statistics should be supplemented with social and human indicators rooted in the basic needs approach. This approach was applied by the International Labour Organization in its 1976 report which framed economic growth as only one part of a larger development goal (International Labour Office 1976). In 1977, Leipziger and Lewis then proposed a new measurement approach for assessing development policies based on basic needs (Leipziger and Lewis 1977). Around the same time, Morris (1978) proposed a comparable international index to measure development, the Physical Quality of Life Index (PQLI), the precurser to the Human Development Index. Since the start of the 2000s, critical advances have been made in the measurement of multidimensional deprivation and poverty, both at micro-level (Alkire and Foster 2011; Alkire et al. 2015; Atkinson 2003; Bourguignon and Chakravarty 2003; Gordon et al. 2003), and at macro-level, with the introduction of composite indexes, such as the Human Development Index (HDI), introduced by the United Nations Development Programme in 1990, arguably the most successful effort to rank countries on non-monetary outcomes.

Despite some criticism of multidimensional and composite indexes (Ravallion 2011; Greco et al. 2019; Biggeri et al. 2019), the international community explicitly recognize the important role of multidimensional measures of poverty, and the fact that poverty affects groups of the population differently. The Sustainable Development Goals (SDGs) include a specific mention of poverty “in all its dimensions” in target 1.2, and more recently, the Atkinson commission on Global Poverty endorsed the measurement of multidimensional poverty, along with monetary poverty, to track progress towards SDG 1.2 (World Bank 2017).

Multidimensional deprivation indices measure the simultaneous occurrence of multiple deprivations in an individual or household. Any attempt to reduce multidimensional deprivation should therefore, in principle, improve lives in multiple domains of wellbeing at the same time. Programs and policies that lead to improved outcomes in multiple but separate domains, however, do not automatically yield an impact on multidimensional poverty—they need to impact the same people in multiple ways in order to succesfully reduce multidimensional poverty (Duclos and Tiberti 2016). For this reason, the use of multidimensional poverty measures for the evaluation of for any single intervention, program, or policy sets a higher bar for success since interventions need to address multiple deprivations to have impact, not only by reducing each deprivation on its own, but also lowering the probability of their simultaneous occurrence. For instance, an intervention that both increases educational attendance and reduces the need for children to work by providing financial support to families can break the link between low school attendance and child labour, reducing the chances of either event occurring.

Individuals who experience multiple deprivations at the same time are particularly vulnerable to additional economic and social hardships, so it is crucial to assess the impacts of interventions on multidimensional deprivations, especially social protection policies, which are important tools for poverty reduction in developing countries. One issue with common measures of poverty, both monetary and multidimensional, is that they are most often household-based and overlook intra-household allocation, which can result in an underestimation of gender inequality among the poor (Espinoza-Delgado and Klasen 2018). We address this issue directly in this paper by creating a novel individual-level measure for multidimensional deprivation among young women in South Africa and examining the impact of a conditional cash transfer program on this unique measure. Girls and young women, particularly those living in poor, rural areas are acutely vulnerable to multiple deprivations and are at a greater risk of a lifetime of poverty because they are likely to be found at the intersection of different disadvantages including the burden of nonequitable gender norms, low socio-economic status, and young age. While we know that cash transfer programs can have a positive impact for girls across a number of separate domains, understanding whether programs can improve their lives in a comprehensive way is a step forward towards building the evidence for social protection programs not only as a tool to improve wellbeing in the short-term, but as a way to improve transitions to adulthood.

Our paper’s main aim is to help fill the evidence gap surrounding the effect of social protection interventions on multidimenional poverty, through defining and employing an individual-level measure of wellbeing for young women. To our knowledge, no study so far has addressed the impact of an intervention on the multidimensional poverty status of a specific population, especially adolescent girls and young women. We use primary data from HIV Prevention Trials Network (HPTN) 068, or Swa Koteka, an experimental intervention designed to test the efficacy of cash transfers, conditional on school attendance, for HIV prevention among adolescent girls and young women in South Africa. Analyzing longitudinal data from the conditional cash transfer (CCT) intervention, we seek to understand the impact of the program on the multidimensional nature of wellbeing for young women using an index measure that captures multiple individual-level deprivations. We find that the program reduced multiple deprivations for participants thereby improving wellbeing and reducing the experience of multidimensional poverty for these young women.

## Background

Social protection, part of the Sustainable Development agenda itself (SDG target 1.3), and cash transfers in particular, have shown to be powerful instruments to address several important development objectives like reduction of household poverty, greater food insecurity, and improved child schooling outcomes (Bastagli et al. 2016). The South African Child Support Grant (CSG), for instance, has been shown to improve child nutrition and food security of households (Coetzee 2013); lower risk of mental health disorders (Plagerson et al. 2011); and improve school attendance among adolescents through lowering the associated material and psychosocial costs (Adato et al. 2016). Moreover, it is clear that cash transfer programs, which are often intended as a social safety net for the poorest households, reduce monetary poverty, especially for the some of the members who are more likely to be vulnerable to economic distress, such as children. Findings have also shown that the South African CSG is effective in reducing childhood poverty (Barnes et al. 2017). In a review of the effect of cash transfers on childhood poverty across different settings (Sub-Saharan Africa, Latin America, and transition economies), Barrientos and DeJong (2006) find that overall, cash transfers have a positive impact, regardless of whether they are conditional or unconditional cash transfers.

Interventions targeted to women and girls may also contribute to additional development goals of improving gender inequality (SDG 5) through means such as reducing intimate partner violence (SDG 5.2.1) (Buller et al. 2018; Kilburn et al. 2018). In some cases, including the study we report on here, cash transfers have even been designed with the objective of reducing new HIV infections among young women and other vulnerable populations (SDG 3.3.1), but these interventions have not shown great promise in meeting this objective (Stoner et al. unpublished; de Walque et al. 2012; Pettifor et al. 2012, 2016a). However, a similar cash transfer intervention for young women in Malawi, while not designed with an HIV prevention objective, found a reduced prevalence of HIV and HSV-2 infection among girls who were attending school at baseline (Baird et al. 2012).

Across Sub-Saharan Africa (SSA), national cash transfer schemes, for the purposes of social protection, have also been effective in improving related health behavior outcomes among young people including sexual risk behaviors, child pregnancy, and early marriage (Owusu-Addo et al. 2018). Evidence from Kenya’s national cash transfer scheme shows that it had protective effects for young people, reducing the likelihood of first pregnancy of females 15–25 (Handa et al. 2015), and delaying sexual debut for both males and females 12–24 (Handa et al. 2017). Additionally, the program was found to reduce depressive symptoms among youth, especially for young men 20–24 (Kilburn et al. 2016). The South Africa CSG, has also been shown to mitigate the risk of HIV infection in adolescents by reducing risky behaviors such as early sexual debut (Heinrich et al. 2017; Cluver et al. 2016).

On the other hand, the effects of cash transfer schemes on gender relations and women’s empowerment is mixed. Earlier evidence from Latin America shows that cash transfers targeted to women do not necessarily increase their power over household resources (Handa et al. 2009), and a systematic review finds no definitive evidence that cash transfers increase women’s decision-making power (Yoong et al. 2012). More recently, qualitative evidence from Zambia indicates that a cash transfer program improved feelings of empowerment among women, but quantitative evidence suggests that it only had a modest effect on women’s decision-making power due to strongly held gender norms (Bonilla et al. 2016). Additionally, Patel and Hochfeld (2011) find that while the South Africa’s CSG program improved women’s ability to control resources, it did not lower the burden of care for women in the household, even in the face of better employment opportunities for women outside the household.

Despite the strong evidence that cash transfers can improve many individual aspects of wellbeing, evidence is lacking on the effect of these social protection interventions on multidimensional deprivation, either at the individual or household-level, and both in low and higher income settings. Among the few studies that analyze the impact of interventions on multidimensional outcomes, Chowdhury and Mukhopadhaya (2012) use a multidimensional poverty framework to assess the effectiveness of NGOs and governments’ microfinance programs in Bangladesh. They find that in many dimensions, government-based interventions are more efficient than NGO’s. Their results, however, are not focused on the reduction of poverty, but rather on the dimensions impacted by the program. In Bangladesh, Robano and Smith (2014) find that a NGO-run anti-poverty program (involving transfers of physical assets and information) led to a substantial reduction in multidimensional poverty. Among the evidence from higher-income settings, Notten and Guio (2016) find considerable effects of cash transfers on household-level material deprivation in five EU countries using income elasticity to indirectly estimate the impacts.

The relationship between multidimensional deprivation and gender is not new in the literature. Batana (2013) constructs a specific index of multidimensional poverty for women in sub-Saharan Africa, using four dimensions: assets, health, education, and empowerment. The author finds that adding specific dimensions for women in a multidimensional measure changes the ranking of countries, with respect to other more standard measures, such as the Human Development Index. Rogan (2016), on the other hand, investigates the level of multidimensional poverty of women in South Africa, using the Global Multidimensional Poverty Index, and finds that the multidimensional gender poverty gap is quite similar to the monetary gender poverty gap; female headed households are more likely to be multidimensionally poor. However, the study does not use any dimension specific to women, nor does it use an individual-based index.

Other approaches, such as Vijaya et al. (2014) have constructed individually-based measures of multidimensional poverty in order to better capture and measure the gender differences in multidimensional poverty. Additionally, the Women’s Empowerment in Agriculture Index (WEAI) was developed by the International Food and Policy Research Institute (IFPRI) and the Oxford Poverty and Human Development Initiative (OPHI) as an extension of the Alkire and Foster (2011) method to specifically measure the empowerment and decision-making power of women in the agricultural sector. The WEAI measures the empowerment of women in five domains: production, resources, income, leadership, and time. We expand this literature, developing a measure of multidimensional deprivation that is specific to both individual age and gender.

## Framework

To understand the channels through which a conditional cash transfer program can affect the multidimensional deprivation of young women, we apply an expanded socio-ecological framework (Biggeri et al. 2018; Biggeri and Ferrannini 2014; Yousefzadeh et al. 2019). The underpinning of our framework is similar to a ‘bottom-up’ model of subjective wellbeing (Andrews and Withey 2012; Headey et al. 1985). The cash transfer affects the dimensions of deprivation, which results in a lower deprivation.

**Fig. 1:**
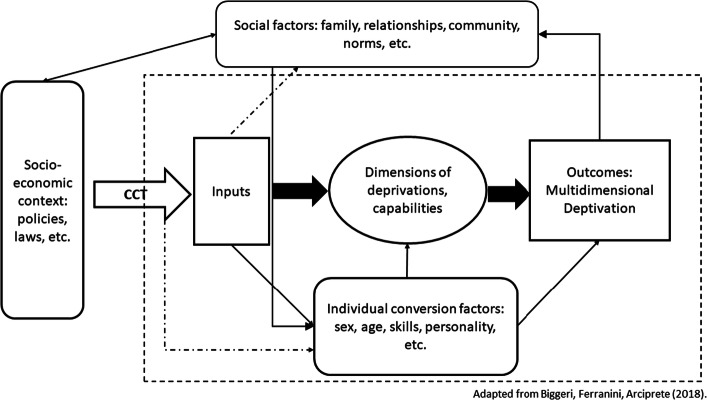
Theoretical framework describing the pathway of influence of a cash transfer intervention on young women’s multidimensional deprivation

This framework was first introduced by Bronfenbrenner to model child development (Bronfenbrenner 1979; Bronfenbrenner and Morris 1998). It frames the individual within a series of concentric systems, from the smallest one (the individual) to the macro-systems shaping the broader society, passing through intermediate systems such as the family and the local context. We adapt it to our context following Biggeri et al. (2018), incorporating Sen’s capability approach (Sen 1985). Figure 1 exemplifies how the cash transfer enters the system, and how it can not only affect outcomes, but other parts of the system itself.

In our case, the conditional cash transfer intervention, which is an external policy from the broader socio-economic context, affects the inputs, increasing the economic means of the girl and her family^1^ (as long as she meets the schooling requirement). This is turn can affect the dimensions of deprivation, and lead to improvements in the outcome of multidimensional deprivation. However, the cash transfer not only influces the material inputs, but can also affect the conversion factors of the individual—a conditional cash transfer which requires girls to stay in school changes the girl’s situation by increasing her education and reducing her exposure to risks. At the same time, the transfer can increase her sense of independence and self worth, which in turn can affect the outcomes. Finally, the outcome itself can affect the girl’s context, in terms of social norms, relationship with peers and family, and that can both affect the conversion factors, and influence the broader society (for example, when interventions are successful, they are more likely to be replicated and expanded).

As detailed above, cash transfers have had success in improving both material conditions of recipients, and non-material conditions, for example lowering psychological distress (Baird et al. 2013) and decreasing intimate partner violence (Heath et al. 2019). They can also affect girls’ freedom of choice, by decreasing the occurrence of early marriage and pregnancy among participants (Baird et al. 2010). Thus, cash transfers not only influence deprivation directly, but they may also improve an individual’s ability to convert input into outcomes, by affecting her independence, her relationships, and her mental and emotional state.

## Data and Methods

### Study Site

This study took place in a rural, poor area of Mpumalanga province, South Africa near the Mozambican border. Participants for this study were recruited from villages within the Agincourt Health and Socio-Demographic Surveillance Systems (HDSS), a demographic monitoring system that has been ongoing since the early 1990s. The Agincourt study area is characterized by high poverty and unemployment. Temporary migration for work is common, not only for young men, but increasingly for young women (Kahn et al. 2012). Farming is not a major source of income or food because of the arid landscape. Therefore, many households are food insecure and rely on government support to get by, particularly South Africa’s non-contributory grant programs like the Old Age Pension and the Child Support Grant (CSG) (Kahn et al. 2012).

Our study sample comprises some of the most poor and vulnerable households both in South African and in the HDSS. Over 80% of study households were receiving the CSG for at least one of their children, indicating official recognition of poverty status since the CSG is designed to be a social support program for children (under the age of 18) living in the poorest households in South Africa. Household consumption of our sample is also much lower compared to the rest of South Africa and most households would be defined as poor by government standards. At the start of baseline data collection in 2011, the official poverty line in South Africa was 620 Rand per capita/month while the average per capita monthly expenditure among our study sample at baseline was only 460 Rand, demonstrating consumption rates well below the poverty line (Stats SA 2014; Kilburn et al. 2019). Food expenditures among our sample also makes up around half of total expenditures signifying that most consumption was for basic needs. Moreover, the young women participants reported high levels of food insecurity at baseline with around a third reporting having been worried about having enough food in the past 12 months (Kilburn et al. 2019). Comparatively, across South Africa, 36% of households were considered poor and 23% were food-poor according to official poverty lines at the time of baseline data collection in 2011 (Stats SA 2017). Young people and particularly females, however, are known to be at increased risk of poverty in South Africa (*ibidem*).

Our study area is also characterized by high HIV prevalence. Peak prevalence from the most recent HDSS HIV prevalence survey in 2010 was 45.3% among men and 46.1% among women, both aged 35–39 (Gómez-Olivé et al. 2013). The same 2010 survey found HIV prevalence at 5.5% among girls aged 15–19 and 27% among young women aged 20–24, highlighting the need to target prevention strategies towards young women, a particularly vulnerable group in SSA (*ibidem*). This evidence was a primary motivation for targeting the prevention intervention to young women in high school before they transitioned to adulthood (Pettifor et al. 2016b). The HPTN 068 trial found incidence among young women during the trial of around 2% (per person-year) (*ibidem*).

### Study Design and Sampling

To test whether CCTs are an effective HIV prevention strategy, HPTN 068 (or Swa Koteka which means “it is possible”), was designed as an individually randomized conditional cash transfer (CCT) intervention for females attending high school in the Agincourt area. It was hypothesized that the intervention would reduce HIV incidence because it would incentivize girls to stay in school and reduce young women’s economic insecurity, both recognized as protective factors for HIV acquisition among young women. Swa Koteka provided monthly cash transfers for up to three academic years to study participants (and their parents or guardians) that were randomized to the treatment group if they attended school at least 80% of school days in the previous month. Attendance was verified with official school records.

Monthly cash transfers amounts were the same for all beneficiaries, 100 Rand for the young women and 200 Rand for the parent or guardian (roughly US$ 10 and US$ 20 using 2012 conversion rates).

The total amount, 300 Rand, was chosen as it approximated the amount per child provided by the CSG at time, the South African social protection program most households in the study were already receiving. Since average per capita monthly consumption for study households was 460 Rand at baseline, the cash transfer represented a significant proportion of household consumption.

Adolescent girls and young women living in the HDSS were recruited to the study beginning in March 2011. Eligibility requirements included being between 13 and 20 years old, enrolled in a participating high school in the study area, able to read, living with at least one parent or guardian, and not married or pregnant. Additionally, participants had to have the appropriate documents to open a bank or post office account in order to receive their transfers. The most common reason girls were ineligible was because they were either not in school or not enrolled in a participating school or an eligible grade (45%); only 2% of girls screened did not meet the requirements for documentation (Pettifor et al. 2016b). After screening procedures were completed within participating high schools in the HDSS, 2537 girls were found eligible and recruited as study participants.

Written informed consent for study participation was obtained at home visits from both young women (unless younger than 18 years old) and her parent or guardian. Written assent was obtained for female participants under 18 years old and written consent was provided from her parent or guardian. Institutional Review Board approval for this study was obtained from the University of North Carolina at Chapel Hill and the University of the Witwatersrand Human Research Ethics Committee as well as the Provincial Department of Health’s Research Ethics Committee.

Once enrolled in the HPTN 068 study, participants completed a baseline survey and were tested for HIV and Herpes Simplex Virus 2 (HSV-2). The survey was self-administered using an Audio Computer-Assisted Self-Interview (ACASI) to elicit more reliable responses to self-reported questions on sensitive topics including sexual behaviors and partner violence. HIV and HSV-2 tests were administered after the ACASI survey and included pre and post-test HIV counselling. In addition, parents or guardians of the young women also completed a household survey that was administered by a researcher.

After all baseline assessments were completed, the study team individually randomized study participants (including her parent or guardian) 1:1 to the intervention (1261 treatment and 1272 control). Participants in the treatment arm would receive the monthly cash transfer (as long as they met the attendance requirement) until either the study ended in 2015 or they graduated high school, whichever came first, while the participants in the control arm would not receive any compensation. At annual intervals after baseline (12, 24, and 36 months), study participants completed the same ACASI survey (and household survey for parents or guardians) and HIV and HSV-2 testing if they tested negative at the prior visit. Attrition across rounds was very low outside of the expected loss of young women that graduated high school (Pettifor et al. 2016a).

### Impacts of the Program

The impacts of the Swa Koteka intervention on several domains have already been studied, including the main outcomes of HIV incidence and sexual risk behaviors (Pettifor et al. 2016a; Kilburn et al. 2018; MacPhail et al. 2017). In the main analysis of HPTN 068, Pettifor and coauthors (Pettifor et al. 2016a) found that the CCT did not lead to an impact on HIV incidence (or HSV-2) among young women, the study’s primary objective. In fact, they found HIV incidence was relatively low across the entire sample at 1.8% per year given their expectation of around 3% (*ibidem*). One reason suggested for the null findings was that school attendance did not differ across treatment and control groups. Young women in both groups attended school at unexpectedly high levels of around 95% even though the cash was conditional on school attendance. As increased school attendance was hypothesized as the main (protective) pathway that would affect HIV risk, the authors believed the high rates of school attendance contributed to their null findings. This was further supported with evidence that HIV incidence did vary between young women who attended at high levels and those who attended at less than 80% or dropped out. Young women who attended school less than 80% of expected time were at increased risk of HIV acquisition, irrespective of study arm (Pettifor et al. 2016a).

While previous analyses of this intervention have focused on evaluating the impact of the CCT on specific domains focused on health and behavior, we look across many different domains in a holistic way. Additionally, we add domains and indicators that have not been analyzed yet.

### Measures of Multidimensional Deprivation

Using data from the ACASI questionnaire, we construct an individual measure of multidimensional deprivation (MDD) for the young women. We focus on individual indicators, which are more specific to our adolescent sample and less dependent on assumptions about household sharing rules and assets. We define two MDD measures: an index measure and a weighted score both derived using the Alkire and Foster (2011) methodology, which involves counting simultaneous deprivations experienced by individuals across different dimensions of poverty. We define six dimensions of deprivation comprised of fifteen indicators (Table 1). The six dimensions of deprivation include education, health and food security, protection, family and social relationships, economic agency, and psychosocial wellbeing. The choice of dimensions and indicators is based on the multidimensional poverty literature, which uses a mix of basic needs and capabilities frameworks (Alkire et al. 2015). We also incorporate a right-based approach, focusing on lack of access to services and lack of realization of young women’s rights to security and agency, including the protection from violence, the right to food security, and to economic agency. The indicators were chosen to be age and gender-relevant, among those that that were available to us in the dataset. Indicators are defined as binary variables, taking value 1 if the individual is deprived, 0 otherwise. Table 1 shows the definition of deprivation for each indicator we use in this analysis.

**Table 1:** MDP components and weights

Dimensions	Indicator	Definition of deprivation (1 = yes)	Weight (total = 1)
Education			0.167
	Educational achievement	Repeated any grades during main trial	0.056
	Attendance	Attended less than 80% of school days during previous month	0.056
	Dropout	Not enrolled or has dropped out during current term	0.056
Health and food security			0.167
	Food secure	Worry about food in past 12 months	0.056
	Access to health services	No access to birth control. Reasons for no access include: too expensive, don’t know where to find, denied from health worker	0.056
	Reproductive health	Was pregnant and/or gave birth before 20	0.056
Protection			0.167
	Violence	Reported any physical intimate partner violence	0.083
	Sexual violence	Reported any sexual violence by anyone	0.083
Family and social relationships			0.167
	Parental relationships	Parenting monitoring scale. Scored below median on the scale	0.056
	Sexual empowerment	Sexual Relationship Power Scale (SRPS). Scored in bottom tercile on scale	0.056
	Gender attitudes	Gender Equitable Men’s Scale (GEMS). Scored in bottom tercile on scale	0.056
Economic agency			0.167
	Employment and work	One or more of the following applies: (1) Engages in paid work if < 15 years old (2) Engages in paid work that puts young women in unsafe or vulnerable position (sex work, selling drugs, working at tavern, and mining) (3) Does housework/chores for > 15 h a week	0.083
	Economic empowerment	Has none of the following: spending money, savings, or bank account	0.083
Psychosocial well-being			0.167
	Mental health	Measured using the Center for Epidemiological Studies-Depression Scale (CES-D). Depressed mood is a score of 16 or higher^a^	0.083
	Future outlooks/hopefulness	Hope score is in bottom quartile	0.083

^a^The CES-D scale was not available at baseline so the Children’s Depression Index (CDI) was used to compare baseline balance for mental health across treatment arms

The MDD Index is constructed by summing all dimensions over their weighted score calculated using the formula below. First, each individual is either deprived, *D*_i_, in any indicator *t*, according to the defined threshold *k*:

$$D_{i} = \left\{ {\begin{array}{ll} 0 \hfill & {if\quad t < k} \hfill \\ 1 \hfill & {if\quad t \ge k} \hfill \\ \end{array} } \right.$$

Across each dimension, an individual’s dimension deprivation score, *S*_id_, is defined as the ratio of the total number of deprivations (i.e. the number of ‘ones’), *D*_*id*_, to the number of indicators in that dimension, *t*_*d*_:

$$S_{id} = \mathop \sum \limits_{i}^{d} \frac{{D_{id} }}{{t_{d} }}$$

For instance, if a girl is deprived in 2 out of the 3 indicators in the education dimension, her score for that dimension is 2/3. Each individual’s score (*S*_*id*_) is therefore between 0 and 1. Finally, the overall deprivation index (MDD Index) for each individual, *DI*_*i*_, is equal to the sum of scores across the six dimensions:

$$DI_{i} = \mathop \sum \limits_{d = 1}^{6} S_{id}$$

Consequently, the highest MDD Index value is a 6 (deprived in all dimensions and indicators) and the lowest value is 0 (not deprived across all dimensions and indicators).

Then, to construct the MDD weighted score, both dimensions and indicators within dimensions are weighted equally, using a system of so called “nested weights” (see Table 1). The main difference is that each dimension’s deprivation score (*S*_*id*_) are first weighted by 1/6 before being summed so that MDD scores range from 1(fully deprived) to 0 (not deprived). The added advantage of this specification is that it allows for us to easily define and test deprivation cutoffs as additional outcome measures. While our measure adds many domains usually left out of conventional measures of deprivation, we cannot expect to exhaust all the dimensions that are relevant to the wellbeing of girls and young women. Additionally, while we have the advantage of having a context-specific measure, this means it is not comparable with international multidimensional poverty measures.

### Measuring Multidimensional Deprivation using MODA

To test the sensitivity of this measure, we replicated the analysis with a different aggregation process using an approach based on the Multiple Overlapping Deprivation Analysis (MODA). MODA is a tool developed by UNICEF to measure multidimensional child poverty (de Neubourg et al. 2012) and based on the previous work of Gordon et al. (2003) and Roelen et al. (2009). MODA applies a counting approach to measure multidimensional poverty, where dimensions of deprivation are simply added using equal weighting. Outcomes constructed using MODA mirror traditional poverty analysis and include: the headcount ratio, the intensity of deprivation, and the adjusted headcount (the product of the first two). Given the defined number of dimensions, an individual is considered deprived (*D*) if the number of dimensions (*d*) in which the child is deprived is equal to or larger than the cutoff point, *k*. This can be defined as:

$$\begin{array}{*{20}l} {D_{ik} = \, 1\quad \, if\quad d \, \ge \, k} \hfill \\ {D_{ik} = \, 0\quad if\quad d \, < \, k} \hfill \\ \end{array}$$

The headcount ratio of deprived individuals (*H*_*k*_) at any cutoff *k* is therefore defined as follows:

$$H_{k} = \frac{{\mathop \sum \nolimits_{n}^{{N_{k} }} D_{ik} }}{N}$$

where *N* is the total of individuals in a given population or group; *D*_*ik*_ are the individuals who are deprived, as defined above; and *N*_*k*_ is the total of individuals who are deprived according to the cutoff *k*.

The main difference from an Alkire–Foster type of multidimensional index is that MODA uses a triple cutoff. Similar to the Alkire–Foster method, indicators across all dimensions are first assigned a deprivation status based on specific indicator cutoffs (see Table 1). In the second step, however, individuals are classified as either deprived (1) or not (0) in each dimension. In the third step, individuals are defined as multidimensionally poor if the number of dimensions in which an individual is deprived is greater than the chosen cutoff.

The second difference comes from the aggregation process of indicators in dimensions. Instead of using a nested-weights system, MODA uses what is known as the union approach, whereby individuals are classified as deprived in a dimension if they are deprived in any indicator of that dimension. The reasons for this choice are twofold and rooted in the rights-based framework that underlies MODA. First, if indicators reflect a right of the individual, we cannot allow them to be substitutes, but they will necessarily be complements. Second, this approach minimizes exclusion error, a choice also rooted in the rights-based framework. The consequence is a measure of multidimensional poverty that is harder to move with a specific programs or policy since a decrease in deprivation in one indicator does not necessarily translate into a reduction in overall deprivation if other indicators in that dimension are unaffected.

We apply this method to our measure, aggregating indicators into each dimension using the union approach and examining the deprivation for three measures: the number of dimensions a young women is deprived and the headcount ratio using both 2 and 3 dimensions as *k*. We argue that finding impacts of the program on this measure constitutes a strong robustness test for the effect of the program on the multidimensional deprivation of young women.

### Empirical Strategy

To measure the impact of the program, we used both the MDD index and score (derived using the Alkire–Foster aggregation method) as continuous variables. We also used a headcount measure resulting from two different cutoffs: an MDD score above one-sixth (0.17) and above one-third (0.33) where higher scores indicate greater deprivation. We chose the first cutoff as it approximates full deprivation in one dimension, while one-third is a frequent choice with Alkire–Foster indicators and here, distinguishes a more severe poverty line so that we can identify the effect on more acute deprivation. We focus on the impact of the intervention on the score and on the number of dimensions (in the case of MODA), which we argue provides a more comprehensive assessment of the total effect. However, we also evaluate the impact on multidimensional deprivation status or headcount, as reducing headcounts is often an important and quantifiable policy goal. Impacts on the intensity of deprivation are not reported but discussed later.

We estimated the total effect of the CCT intervention on our outcomes using an intent-to-treat (ITT) estimator. The linear model displayed in Eq. (1) shows the basic specification, where $CCT_{i}$ is the indicator for treatment, $Y_{it}$ is the outcome of interest at visit t and $\varepsilon_{it}$ is the error.

1 $$Y_{it} = \beta_{0} + \beta_{1} CCT_{i} + \varepsilon_{it}$$

In addition to Eq. (1), which gives us the total ITT effect, we also estimated Eq. (2) to test for moderation of the treatment effect by (monetary) baseline poverty status where $P_{i}$ represents quartiles of per capita household consumption at baseline and $CCT_{i} P_{i}$ is an interaction term between indicators for treatment and baseline consumption quartiles.

2 $$Y_{it} = \beta_{0} + \beta_{1} CCT_{i} P_{i} + \beta_{2} CCT_{i} + \beta_{3} P_{i} + \varepsilon_{it}$$

Using the *p* value on the interaction term from Eq. (2), we tested for significant differential treatment effects by baseline poverty status. We also used the results from Eq. (2) to estimate marginal impacts of the CCT across each quartile of consumption.

We used General Estimating Equation (GEE) models with robust standard errors to account for repeated observations on participants over three follow-up study visits. All models additionally controlled for a young women’s age and household per capita consumption at baseline. Additional analysis included quantile regression to assess impacts of the intervention across deciles of scores ($Y_{it}$) and two sensitivity analyses. One sensitivity analysis applied Eq. (1) on different versions of the index, and the second estimated the impact of the CCT on MODA deprivation measures described above. All analyses were performed using Stata 14.2.

## Results

Table 2 shows some individual and household baseline characteristics of the sample and Table 3 reports baseline prevalence rates of deprivation across the indicators and mean values for MDD outcome measures, both display results separately for treatment and control groups. The last two columns show the difference between treatment and control arms and the *p* value from equality of means tests. Indicators are balanced between treatment and control across all measures of deprivation (no significant differences).

**Table 2:** Baseline balance and summary statistics for key demographic and outcome variables

	Treatment *N *= 1261	Control *N *= 1272	Difference	*p* value (T-C)
	Mean (SD)/Median (IQR), or %			
Demographics				
Age	15.5 (1.7)	15.5 (1.6)	0.0	0.89
Household size	6.2 (2.7)	6.1 (2.6)	0.1	0.42
Ever had sex	27.1	27.7	− 0.6	0.72
Any sexual partner past 12 months	26.2	27.7	− 1.5	0.42
Transactional sex^a^	15.5	17.1	− 1.6	0.60
Older sexual partner (5 + years)^a^	19.9	20.6	− 0.7	0.82
Household SES				
Household monthly per capita consumption (mean Rand)	455.0 (SD 675.3)	472.7 (SD 672.2)	− 17.7	0.51
Household monthly per capita food consumption (mean Rand)	233.6 (SD 467.8)	239.8 (SD 413.5)	− 6.1	0.73
Asset Index (mean, range 0–61)	14.3	14.2	0.1	0.61
Number of grants to the household	2.7 (SD 2.0)	2.7 (SD 1.9)	0.0	0.27
Poorest (bottom half of total per capita consumption)	50.7	49.2	1.5	0.45
Psychosocial wellbeing				
Sexual relationship power scale (0–24)^a^	15.5 (6.1)	15.6 (5.8)	− 0.1	0.79
Hope score (range 0–39)	31.2 (7.2)	31.2 (7.2)	0.0	0.93
Child’s Depression Index 10 item (0–18)	4.5 (3.1)	4.4 (3.0)	0.1	0.46
Young women’s economic resources				
Always had spending money	9.7	11.0	− 1.3	0.28
Engaged in paid work	15.0	17.1	− 2.1	0.15
Savings	24.8	25.2	− 0.4	0.80
Bank or post office account	15.8	16.5	− 0.7	0.63
Ever borrowed money ‘to get by’	23.4	21.5	1.9	0.25
Food worry (young woman, past 12 months)	32.9	35.7	− 2.8	0.14

*p* values based on equality of means tests with robust standard errors

^a^Only for young women who had ever had sex (N = 693)

**Table 3:** Mean values of deprivation indicators among young women at baseline

	Treatment (N = 1261)	Control (N = 1272)	Difference (T-C)	*p* value
	% (unless otherwise stated)			
Schooling				
Any repeated grades	34.75	35.32	− 0.57	0.76
Low attendance (< 80%)	6.67	6.44	− 0.23	0.83
Food and health				
Food worry	35.38	32.67	2.70	0.15
No birth control access	15.72	16.18	− 0.45	0.75
Early pregnant	8.33	8.17	0.17	0.88
Protection				
Physical partner violence	10.06	11.18	− 1.12	0.36
Sex violence	3.38	2.62	0.76	0.26
Relationships				
Low perceived sexual empowerment	11.24	12.53	− 1.29	0.32
Low gender equity attitudes	42.37	42.43	− 0.05	0.98
Low parental monitoring	46.38	47.98	− 1.59	0.42
Psychosocial				
Depressed mood (CDI at baseline)	25.79	25.61	0.17	0.92
Low hope	33.81	36.56	− 2.75	0.15
Economic agency				
No resources	31.76	32.51	− 0.75	0.69
Poor working conditions	16.35	14.59	1.76	0.22
Multidimensional poverty measures				
MDP index (range of 1–6)	1.02	1.05	− 0.03	0.35
MDP score (range of 0–1)	0.17	0.17	0.00	0.35
Score > 1/6 (%)	51.81	50.44	1.37	0.49
Score > 1/3 (%)	10.06	11.02	− 0.96	0.43
Dimensions of deprivation (MODA)				
Education	35.85	37.83	− 1.98	0.30
Food/health	49.76	48.69	1.07	0.59
Protection	12.62	12.81	− 0.19	0.89
Relationships	72.17	73.12	− 0.95	0.59
Psychosocial	46.78	49.80	− 3.03	0.13
Economic agency	43.90	43.30	0.60	0.76

*p* values calculated Wald tests for the equality of means between Treatment and Control. N = 2533

The main effects of the CCT intervention on MDD are shown in Table 4. The ITT estimates show a clear pattern of reduced deprivation for the young women in the treatment group. The MDD Index (range of 1–6) was reduced by 0.17 points (*p* < 0.01), a 16% change from baseline levels of deprivation. Likewise, the weighted MDD score (range between 0 and 1), was reduced by − 0.03 or 17%. The number of young women falling above defined thresholds of the MDD scores (1/6 and 1/3) was similarly impacted by the intervention—participants in the treatment group were significantly less likely to have scores above 1/6 (effect size: − 10 pp, *p *< 0.01) and 1/3 (effect size: − 3 percentage-points (pp), *p *< 0.01).

**Table 4:** ITT estimates of the effect of CCT on MDP measures

	(1) MDP index	(2) MDP score	(3) Score > 1/6	(4) Score > 1/3
	(Range 0–6)	(Range 0–1)	(Yes/no)	(Yes/no)
Intervention	− 0.17***	− 0.03***	− 0.10***	− 0.03***
	(0.03)	(0.00)	(0.02)	(0.01)
Control mean	1.171	0.195	0.539	0.179

Estimates from linear GEE models. Robust standard errors in parentheses. Adjusted for baseline age and log household PCE. Total of 5301 observations collected from three rounds of data (N = 2364)

Significance: ***p < 0.01; **p < 0.05; *p < 0.1

While results in Table 4 demonstrate the total impact of the intervention on multidimensional deprivation, it is not clear whether the effect is driven by certain dimensions or if all dimensions were impacted in the same way. Therefore, we provide impacts on each dimension (scores range 0–1) that comprises our MDD Index in Table 5. All impacts on individual dimensions are in the expected direction (reduction of deprivation), but the primary dimensions that were impacted by the program were Economic Agency (effect size: − 9 pp, *p* < 0.01), followed by Protection (effect size: − 4 pp, *p* < 0.01) and Relationships (effect size: − 2 pp, *p* < 0.05). Economic agency includes indicators of financial inclusion as well as reduction of hours worked—aspects that would predictably be affected by the cash itself. Reductions of deprivation across Relationships and Protection also suggest increases in individual empowerment. As previously mentioned, while attendance was a condition for receipt of the cash transfer, the intervention did not impact the schooling dimension. In fact, young women in the control group attended school at the same high levels as the treatment group (around 95% attending at least 80% of the time).

**Table 5:** ITT estimates of the effect of CCT on dimensions

	(1)	(2)	(3)	(4)	(5)	(6)
	Schooling	Food/health	Protection	Relationships	Psychosocial	Economic agency
Intervention	− 0.01	− 0.01	− 0.04***	− 0.02**	− 0.01	− 0.09***
	(0.01)	(0.01)	(0.01)	(0.01)	(0.01)	(0.01)
Control mean	0.107	0.153	0.204	0.229	0.297	0.181

Estimates from linear GEE models. Robust standard errors in parentheses. Adjusted for baseline age and log household PCE. Total of 5301 observations collected from three rounds of data (N = 2364)

Significance: ***p < 0.01; **p < 0.05; *p < 0.1

Finally, we test the impact of the intervention on the intensity of deprivation, i.e., the impacts on the MDD score and the number of dimensions only among the deprived individuals (according to our chosen cut-offs). We find evidence that the intervention reduced the intensity of deprivation (in number of dimensions) for those deprived in 2 or more dimensions according to MODA (see Table 14 in “Appendix”), but find no impact on intensity using the other cutoffs. While the intervention is effective in reducing the likelihood of being ‘deprived’, it does not improve all dimensions enough to make much of an impact on the intensity of deprivation for the most deprived.

### Additional Analysis

While the above indicates strong average effects, it is likely that not all subgroups were impacted by the program to the same degree. To measure heterogeneity of impacts, we first examined the distribution of impacts across the sample using quantile regression analysis. Additionally, we examined heterogeneity across different socio-economic characteristics: household consumption level at baseline, parents’ education, and the receipt of supplementary household grants.

Results from the quantile regression are shown in Table 6. Across deciles of scores, we find that impacts of the intervention were relatively stable, with a range of − 0.21 to − 0.10. At the bottom decile of scores (least deprived), effect sizes were smallest (− 0.10) indicating that the intervention did not have as large of an effect on MDD on those relatively better off. Nevertheless, impacts of the intervention hovered around − 0.20 for deciles 4 to 9, indicating a mostly constant impact of the intervention across the more deprived girls, with a small peak around the 6th and 7th decile.

**Table 6:** Distribution of the effects of the CCT using quantile regression

Decile	1	2	3	4	5	6	7	8	9
Intervention	− 0.10***	− 0.16***	− 0.16***	− 0.19***	− 0.18***	− 0.21***	− 0.21***	− 0.20***	− 0.19***
	(0.03)	(0.03)	(0.02)	(0.03)	(0.03)	(0.03)	(0.04)	(0.05)	(0.06)

Estimates from linear quantile regression models. Bootstrapped standard errors in parentheses. Adjusted for baseline age and log household PCE. Total of 5301 observations collected from three rounds of data (N = 2364)

Significance: ***p < 0.01; **p < 0.05; *p < 0.1

Since the transfer was not a poverty-targeted program (even though all young women were of low socio-economic status), we first examined heterogeneity by relative poverty using baseline consumption levels measured in quartiles of monthly per capita household expenditures. Results revealed no significant effect on the interaction term between the intervention and consumption quartiles (results not shown). However, we find that marginal effects, estimated at each quartile of baseline consumption, get steadily larger starting from the 4th quartile (greatest baseline consumption) to the 1st quartile (lowest baseline consumption) (Table 7).

**Table 7:** Marginal effects of CCT on MDP measures by quartiles of baseline PCE

	(1)	(2)	(3)	(4)
Consumption quartile	4th quartile	3rd quartile	2nd quartile	1st quartile
MDP index	− 0.11**	− 0.15***	− 0.20***	− 0.23***
	(0.05)	(0.05)	(0.06)	(0.06)
MDP score	− 0.02**	− 0.03***	− 0.03***	− 0.04***
	(0.01)	(0.01)	(0.01)	(0.01)
Score > 1/6	− 0.10***	− 0.08***	− 0.07**	− 0.14***
	(0.03)	(0.03)	(0.03)	(0.03)
Score > 1/3	− 0.02	− 0.02	− 0.05*	− 0.05*
	(0.02)	(0.02)	(0.02)	(0.03)

Marginal effects estimated from GEE models. Adjusted for baseline age and log household PCE. Total of 5301 observations collected from three rounds of data (N = 2364). Robust standard errors in parentheses

***p < 0.01; **p < 0.05; *p < 0.1

The effects are largest for the lowest consumption quartile (− 0.23 MDD Index, − 0.04 MDD Score, − 14 pp for Score > 1/6, and − 5 pp Score > 1/3) demonstrating that young women who came from the least well-off households at baseline benefited the most from the intervention. Although we do not find a significant interaction effect in the model, these results suggest a relationship between multidimensional deprivation and monetary poverty whereby there are increasing returns from the intervention as household monetary poverty increases. Such relationships have been observed in static simulations, where multidimensional poverty measures are found to be more reactive to an increase in consumption for lower level of consumption or expenditures (see UNICEF Tanzania 2016; or UNICEF Malawi 2016).

We then examined the interaction between the CCT and parents’ level of education on the grounds that parental education is found to be one of the strongest correlates with multidimensional child poverty across sub-Saharan Africa (de Milliano and Plavgo 2018). We tested the interaction between the CCT and separate indicators for fathers and mothers completion of at least primary schooling. In this case, however, education does not appear to perform a relevant role as we find no interaction effects (see Table 11).

Lastly, we examined the interaction between the CCT intervention and the number of additional social support grants the household received. A large majority of households (85%) received at least one other grant, the vast majority of which were CSG grants. Therefore, we tested interactions both with the number of total grants and CSG grants, coded categorically (0, 1–3, 4–6, 7 +), and find that more grants in the household generally increases the impact of the intervention (see Tables 12 and 13 in “Appendix”). Results show that interaction effects increase in size with more social grants, but that alone, the CCT intervention still has a strong impact. Significant interaction effects are mostly limited to the 4 to 6 grant category, likely due limited sample numbers above this threshold. We observe similar effects across MDD outcome variables, although using the higher cutoff of 1/3, we no longer find a significant impact of the CCT intervention, possibly because there could be a high degree of overlap between household grant receipt and acute deprivation defined by this cutoff. Other results show that alone, having more household grants predict higher deprivation scores for young women (control group effects), suggesting that the number of social grants is related to poorer living conditions that affect young women’s wellbeing. Overall, it appears that additional household grants can help to further reduce deprivation of young women, but that even without other grants, the cash still had a strong and independent impact on multidimensional deprivation.

### Sensitivity Analysis

As a robustness check, we performed the ITT analysis using different versions of the MDD Index to assess the sensitivity of the measure to its specifications. In each column in Table 8, we estimated Eq. (1) using three different versions of the MDD Index, each one constructed by taking out a particular dimension that was most impacted by the program (economic agency, protection, or relationships). Results are be consistent with our main findings and indicate a positive impact of the intervention in reducing multidimensional deprivation of young women (Table 8). The weaker impact on the iteration of the index without Economic Agency gives further evidence that economic empowerment of young women is a major pathway of the program’s effect.

**Table 8:** Sensitivity tests using different MDP measures

	(1)	(2)	(3)
	Without economic agency	Without protection	Without relationships
Intervention	− 0.08***	− 0.13***	− 0.16***
	(0.03)	(0.02)	(0.02)
Control mean	0.990	0.966	0.942

Estimates from linear GEE models. Each column shows the effect of CCT on the MDP index after adjusting the index by removing the specified dimension and reweighting. Adjusted for baseline age and log household PCE. Total of 5301 observations collected from three rounds of data (N = 2364). Robust standard errors in parentheses

***p < 0.01; **p < 0.05; *p < 0.1

Finally, we present results from the MODA specification we defined earlier. We again find that our results are robust to this specification and that the intervention reduces multidimensional deprivation of young women (Table 9). The transfer has a consistent effect in reducing both the number of dimensions by almost a third of a dimension and the proportion of girls deprived in more than 1 dimensions and in more than 2 dimensions (by 10 and 9 pp, respectively).

**Table 9:** Effect of CCT on MODA measures

	(1)	(2)	(3)
	N dimensions deprived	Deprived > 1 dimension	Deprived > 2 dimensions
Intervention	− 0.31***	− 0.10***	− 0.09***
	(0.05)	(0.02)	(0.02)
Control mean	2.362	0.688	0.439

Estimates from linear GEE models. Adjusted for baseline age and log household PCE. Total of 5301 observations collected from three rounds of data (N = 2364). Robust standard errors in parentheses

***p < 0.01; **p < 0.05; *p < 0.1

## Discussion

Recently, a considerable body of evidence from sub-Saharan Africa has shown how cash transfer schemes can substantially reduce monetary poverty (Daidone et al. 2017; Handa et al. 2018b), even generating a multiplier effect in the local economy (Handa et al. 2018a). Previous evidence, mainly from Latin America, has stressed the role of conditional cash transfers in reducing monetary poverty (Stampini and Tornarolli 2012; World Bank 2009). There is also substantial evidence that social protection schemes can have impacts on different domains of economic and human development outcomes: from increases in household production, agricultural investments, school enrollment, and decreases in food insecurity (see for example Davis et al. 2016) to intimate partner violence (Buller et al. 2018). However, these outcomes are usually analyzed separately rather than as a multiple deprivation or multidimensional poverty index, even when they are analyzed simultaneously.

In this analysis, we examined the effect of a conditional cash transfer intervention, Swa Koteka, on the young women’s wellbeing in a poor area of South Africa through an individual measure of multidimensional deprivation. We find evidence that this targeted cash transfer program can have wide-ranging impacts on the life of beneficiaries beyond the intended scope as a HIV prevention intervention. Our results demonstrate that the transfer was successful in reducing multidimensional deprivation of the young women and that these effects were robust to different definitions of multidimensional deprivation. We also find that the transfer operates mainly through the channel of increased economic agency, a decrease in experienced physical violence, and an improvement of relationships. While not the traditional domains of poverty analysis, these are all important domains in the life of young women, and contribute to her broader sense of wellbeing. Therefore, we demonstrate that even a targeted intervention was able to improve the wellbeing of the beneficiaries beyond the scope of HIV prevention by decreasing the likelihood of being deprived in multiple domains.

The Swa Koteka intervention provided cash payments to both girls and their guardians, conditional on school attendance, and also provided testing and counseling components once a year when girls underwent HIV/HSV-2 testing. Behind this intervention design was the theory that schooling was protective for HIV risk, but also that women’s economic security and empowerment are strongly interconnected, a link that was demonstrated at baseline among young women enrolled in this study (Jennings et al. 2017). In particular, Jennings et al. (2017) showed that among sexually active young women, having greater economic resources in the form of individual-level resources, like savings and spending money, was associated with safer sexual behaviors (Jennings et al. 2017). In South Africa, HIV-risk behaviors are tightly related to experiences of IPV and power imbalances in sexual relationships (Teitelman et al. 2016). Economic imbalances between men and women play a major role in these conditions, and therefore, the importance of economic empowerment for young women is key in addressing risk behavior and reducing HIV infections in young people (Luke 2003). Although Swa Koteka included educational and testing components, the intervention did not effect school attendance or HIV incidence, therefore, its main operating influence on participant wellbeing was likely through the monthly cash payments (allocated in accordance with the attendance requirement).

Our findings align with that theory that individual economic empowerment, provided through mechanisms such as cash transfers, can lead to increased wellbeing for young women across a range of outcomes. Similar to our results here, findings from previous studies have shown that interventions that improve the individual economic opportunities available to poor young women, can have positive impacts on beneficiaries economic agency, behavior, and sense of empowerment. This includes evidence from the Zomba trial, a similar cash transfer intervention for adolescent girls in Malawi, which found improvements for girls across a wide range of outcomes. In particular, the intervention led to increased levels of schooling, reduced HIV prevalence and other sexual risk behaviors, and improved mental health (Baird et al. 2010, 2012, 2013). By examining outcomes of the intervention together as part of measure of multidimensional deprivation, this analysis helps build on this evidence to establish a causal link between targeted social protection programs and young women’s holistic wellbeing.

We also find that deprivation was further reduced for young women receiving the cash transfer when the household also received more grants, suggesting that household income is still an important component of wellbeing of the young women. However, the intervention’s impact was robust even in the absence of household grants, which speaks to the fact that the structure and targeting of the transfer was also important for reducing deprivation at the individual-level. Since the program was targeted to young women and a portion of the cash transfer was allocated directly to the young women every month, this likely had a greater impact on participants wellbeing than a universal increase in household income from government grants. This demonstrates how household-level measures of poverty, both monetary and multidimensional, are likely to mask intra-household allocations and inequalities. However, with a novel measure of individual multidimensional wellbeing for young women we find that targeted cash payments can improve a range of outcomes for individuals who have traditionally been socially and economically without power.

This analysis is strengthened by its strong experimental study design, longitudinal data, and range of unique individual measures. Due to data limitations, however, our multidimensional measure of deprivation inevitably does not include every dimension of wellbeing nor do the dimensions we examine exhaust all of the potential deprivations in that category. Since the observed effect of any intervention or policy is crucially related to the construction of the measure itself, it is critical to perform sensitivity and robustness tests. In this work, we performed different robustness checks to test the validity of the results. First, we used two alternative ways to aggregate indicators into the final measure of multidimensional deprivation, and then for each, we defined two different cutoff points. Second, we modified the original measure by separately excluding dimensions of economic agency, protection, and relationships. We performed the analysis again on each of these adjusted measures and found consistent results.

The results observed here are relevant for policy-makers. Social development ministries could use this evidence to design multidimensional poverty measures that are specific to young people, and can capture their vulnerabilities. However, it will be important to take care when deciding what to include in these measures across different contexts. The dimensions that compose a multidimensional poverty measure can be more or less sensitive to households’ monetary resources, while they can also depend on a vast array of factors, including supply-side constraints (e.g. schooling, healthcare, housing, water and sanitation, or telecommunications infrastructure). A broader definition of poverty that includes non-material dimensions of deprivation, such as the one included here with dimensions including psychosocial wellbeing, protection, and relationships, will depend on social norms, culture, and institutions more than monetary means.

Given this context, detecting impacts of cash transfers and other social protection policies on multidimensional deprivation and poverty will be harder to detect than impacts on ‘conventional’ outcomes, such increased schooling of children or increased spending on productive activities. Nonetheless, as the scope of social protection programs widens in sub-Saharan Africa, and especially the use of cash transfer, evaluations should make use of multidimensional poverty measurement tools to fully assess the simultaneous impacts of programs across a wider range of outcomes that affect wellbeing of beneficiaries.

## Conclusions

This study is one of the first to evaluate the impacts of a social protection intervention on an adapted measure of multidimensional poverty and provides strong evidence that cash transfers reduce multiple forms of deprivation for young women. Findings indicate that interventions targeted towards vulnerable populations for a specific goal can still be as effective as social protection tools that work to reduce poverty in all its forms, helping achieve target 1.2.2 of the SDGs.

Across sub-Saharan Africa, social protection and targeted interventions that provide cash assistance and economic empowerment for young women may have the greatest potential to significantly improve the wellbeing of beneficiaries in the same comprehensive way, resulting in both immediate and long lasting effects. Although evidence on individual, multidimensional wellbeing is lacking, studies in South Africa have shown that the South African CSG, similar to this intervention, has improved many adolescents outcomes, especially young women’s sexual behaviors and psychosocial wellbeing (Cluver et al. 2013; 2014). As have other social protection programs in Malawi, Kenya, and Zimbabwe (Angeles et al. 2019; Handa et al. 2014, 2015; Zimbabwe Harmonised Social Cash Transfer Evaluation Team 2018). Moreover, evaluations of other economic empowerment interventions for youth across sub-Saharan Africa, including many that are for HIV prevention, have shown reductions in violence and changes in sexual behavior for girls that suggest that interventions like these across other contexts can lead to greater empowerment for women and may improve multidimensional wellbeing in the same way (Baird et al. 2010, 2012, 2013; Nyqvist et al. 2018; de Walque et al. 2012).

The numbers of youth will continue increasing in the African continent throughout the century, with the population of 15–24 years old expected to double by 2055 from its 2015 levels (UN Population Division 2015). To harness their potential, countries need to implement policies that best address their needs and support their safe transition to adulthood. The results from this analysis show that a cash transfer intervention improved wellbeing across a range of dimensions for one of the most vulnerable youth populations—poor adolescent girls and young women. Programs and policies that reach this population are critical for meeting important development goals including reducing gendered social and economic imbalances, improving access to quality education, and promoting the prevention of health risk behaviors, that all help create lasting, generational impacts on wellbeing. While cash transfers are not the only tools to reduce multidimensional deprivation for young women, our results clearly show that they can provide a viable option. Policies that combine cash transfers with other interventions that address non-material components of wellbeing, will have the greatest potential to reduce poverty in all its forms, and can be a strategic tool to address the needs of adolescent girls and young women.

## Appendix

See Tables 10, 11, 12, 13 and 14.

**Table 10:** Effect of CCT on individual indicators

	CCT	Control mean
Any repeated grades	− 0.01	0.21
	(0.01)	
Low attendance (< 80%)	0.00	0.05
	(0.01)	
Not in school	− 0.01	0.06
	(0.01)	
Food worry	− 0.01	0.25
	(0.01)	
No access to birth control	− 0.00	0.06
	(0.01)	
Early pregnancy	− 0.02	0.15
	(0.01)	
Physical partner violence	− 0.09***	0.27
	(0.01)	
Sex violence	0.01	0.14
	(0.01)	
Low power	− 0.02	0.17
	(0.01)	
Low gems	− 0.01	0.10
	(0.01)	
Low parenting	− 0.02	0.42
	(0.02)	
Depressed	− 0.02	0.29
	(0.01)	
Low hope	0.00	0.30
	(0.02)	
No resources	− 0.18***	0.26
	(0.01)	
Poor working conditions	− 0.00	0.10
	(0.01)	

Estimates from linear GEE models. Adjusted for baseline age and log household PCE. Total of 5301 observations collected from three rounds of data (N = 2364). Robust standard errors in parentheses

****p* < 0.01; ***p* < 0.05; **p* < 0.1

**Table 11:** Interaction of CCT with parents’ education

	MDP index		MDP score		MDP > 1/3		MD poor > 1/6	
	Father	Mother	Father	Mother	Father	Mother	Father	Mother
Intervention	− 0.23***	− 0.20***	− 0.04***	− 0.03***	− 0.06***	− 0.04**	− 0.12***	− 0.11***
	(0.05)	(0.05)	(0.01)	(0.01)	(0.02)	(0.02)	(0.03)	(0.03)
Education: completed primary schooling	− 0.11**	− 0.09**	− 0.02**	− 0.02**	− 0.04*	− 0.02	− 0.03	− 0.04
	(0.05)	(0.04)	(0.01)	(0.01)	(0.02)	(0.02)	(0.03)	(0.02)
Intervention# completed primary schooling	0.08	0.05	0.01	0.01	0.04	0.01	0.03	0.02
	(0.06)	(0.06)	(0.01)	(0.01)	(0.03)	(0.03)	(0.04)	(0.03)
Observations (N)	4178 (1978)	4796 (2256)	4178 (1978)	4796 (2256)	4178 (1978)	4796 (2256)	4178 (1978)	4796 (2256)

Estimates from linear GEE models. Adjusted for baseline age and log household PCE. Robust standard errors in parentheses

****p* < 0.01; ***p* < 0.05; **p* < 0.1

**Table 12:** Interaction of CCT and number of grants received by the household

	MDP index	MDP score	MD poor: > 1/3	MD poor: > 1/6
Original effect	− 0.17***	− 0.03***	− 0.03***	− 0.10***
	(1)	(2)	(3)	(4)
Intervention	− 0.10*	− 0.02*	− 0.01	− 0.07*
	(0.06)	(0.01)	(0.03)	(0.04)
1–3 grants	0.10**	0.02**	0.02	0.05*
	(0.04)	(0.01)	(0.02)	(0.03)
4–6 grants	0.23***	0.04***	0.06	0.12***
	(0.08)	(0.01)	(0.04)	(0.05)
7+ grants	0.05	0.01	− 0.07	0.18**
	(0.14)	(0.02)	(0.08)	(0.09)
Interactions (reference group: 0 grants)				
Intervention # 1–3 grants	− 0.06	− 0.01	− 0.03	− 0.02
	(0.06)	(0.01)	(0.03)	(0.04)
Intervention # 4–6 grants	− 0.26**	− 0.04**	− 0.05	− 0.16**
	(0.10)	(0.02)	(0.05)	(0.06)
Intervention # 7+ grants	− 0.27	− 0.04	0.05	− 0.36***
	(0.21)	(0.03)	(0.11)	(0.13)

Estimates from linear GEE models. Adjusted for baseline age and log household PCE. Total of 5031 observations collected from three rounds of data (N = 2364). Robust standard errors in parentheses

****p* < 0.01; ***p* < 0.05; **p* < 0.1

**Table 13:** Interaction of CCT and number of Child Support Grants (CSGs) received by the household

	MDP index	MDP score	MD poor: > 1/3	MD poor: > 1/6
Original effect	− 0.17***	− 0.03***	− 0.03***	− 0.10***
	(1)	(2)	(3)	(4)
Intervention	− 0.12**	− 0.02**	− 0.02	− 0.06**
	(0.05)	(0.01)	(0.02)	(0.03)
1–3 grants	0.07**	0.01**	0.02	0.03
	(0.04)	(0.01)	(0.02)	(0.02)
4–6 grants	0.20***	0.03***	0.06	0.11**
	(0.07)	(0.01)	(0.04)	(0.05)
7+ grants	0.01	0.00	− 0.07	0.16*
	(0.13)	(0.02)	(0.08)	(0.09)
Interactions (reference group:0 grants)				
Intervention # 1–3 grants	− 0.06	− 0.01	− 0.02	− 0.03
	(0.05)	(0.01)	(0.02)	(0.03)
Intervention # 4–6 grants	− 0.31***	− 0.05***	− 0.07	− 0.22***
	(0.10)	(0.02)	(0.05)	(0.07)
Intervention # 7+ grants	− 0.25	− 0.04	0.06	− 0.37***
	(0.20)	(0.03)	(0.11)	(0.13)

Estimates from linear GEE models. Adjusted for baseline age and log household PCE. Total of 5031 observations collected from three rounds of data (N = 2364). Robust standard errors in parentheses

****p* < 0.01; ***p* < 0.05; **p* < 0.1

**Table 14:** Effect of CCT on intensity of deprivation

	(1)	(2)	(3)	(4)	(5)
	Intensity—deprived 1/3	Intensity—deprived 1/6	Intensity—Md gap (1/3)	Intensity—Moda 2+	Intensity—Moda 3+
Intervention	− 0.00	− 0.01	− 0.01	− 0.11***	− 0.04
	(0.01)	(0.00)	(0.02)	(0.04)	(0.04)
Observations	795	2453	795	3227	1982
Number of uid	593	1538	593	1870	1313
Control mean	0.427	0.297	0.297	3.122	3.763

Robust standard errors in parentheses

****p* < 0.01; ***p* < 0.05; **p* < 0.1

## Footnotes

1 This is similar to a standard economic model: the transfer relaxes the budget constraint of the family.
